# SFEF-Net: Scattering Feature Extraction and Fusion Network for Aircraft Detection in SAR Images

**DOI:** 10.3390/s25102988

**Published:** 2025-05-09

**Authors:** Qiang Zhou, Zongxu Pan, Ben Niu

**Affiliations:** 1 Aerospace Information Research Institute, Chinese Academy of Sciences, Beijing 100094, China; zhouqiang21@mails.ucas.ac.cn (Q.Z.); niuben@aircas.ac.cn (B.N.); 2 Key Laboratory of Technology in Geo-Spatial Information Processing and Application System, Chinese Academy of Sciences, Beijing 100190, China; 3 School of Electronic, Electrical and Communication Engineering, University of Chinese Academy of Sciences, Beijing 100049, China

**Keywords:** deep learning, global information fusion and distribution, noise-robust loss, scattering feature extraction, synthetic aperture radar, aircraft detection

## Abstract

Synthetic aperture radar (SAR) offers robust Earth observation capabilities under diverse lighting and weather conditions, making SAR-based aircraft detection crucial for various applications. However, this task presents significant challenges, including extracting discrete scattering features, mitigating interference from complex backgrounds, and handling potential label noise. To tackle these issues, we propose the scattering feature extraction and fusion network (SFEF-Net). Firstly, we proposed an innovative sparse convolution operator and applied it to feature extraction. Compared to traditional convolution, sparse convolution offers more flexible sampling positions and a larger receptive field without increasing the number of parameters, which enables SFEF-Net to better extract discrete features. Secondly, we developed the global information fusion and distribution module (GIFD) to fuse feature maps of different levels and scales. GIFD possesses the capability for global modeling, enabling the comprehensive fusion of multi-scale features and the utilization of contextual information. Additionally, we introduced a noise-robust loss to mitigate the adverse effects of label noise by reducing the weight of outliers. To assess the performance of our proposed method, we carried out comprehensive experiments utilizing the SAR-AIRcraft1.0 dataset. The experimental results demonstrate the outstanding performance of SFEF-Net.

## 1. Introduction

Synthetic aperture radar (SAR), as an active microwave technology, provides the ability to observe the Earth’s surface around the clock and in any weather [1]. Aircraft are an important category of objects in SAR images, and they have significant applications in many aspects [2], for example, civil aviation airport management, disaster emergency response, etc. As a result, detecting aircraft in SAR images has become a focal point of research over the past years [3,4].

Traditional SAR automatic target detection methods are usually based on handcrafted features, which have limited generalization capabilities. Constant false alarm rate (CFAR) is the most classical traditional detection method, which uses the threshold and clutter statistics approach [5]. Recent years have witnessed rapid progress in deep learning algorithms, which have excelled in a wide range of tasks, such as image classification and object detection [6,7,8,9,10,11,12,13,14]. Some researchers have started to apply deep learning to the field of object detection in SAR images. Wang et al. [15] designed an aircraft detection and recognition method combined with scattering perception to address the problem of complex backgrounds, but their method’s performance is still sub-optimal. Zhao et al. [16] proposed a pyramid attention dilated network (PADN) to enhance the relationship among the discrete backscattering features of aircraft. However, their method lacks robustness against complex backgrounds.

**Figure 1:**
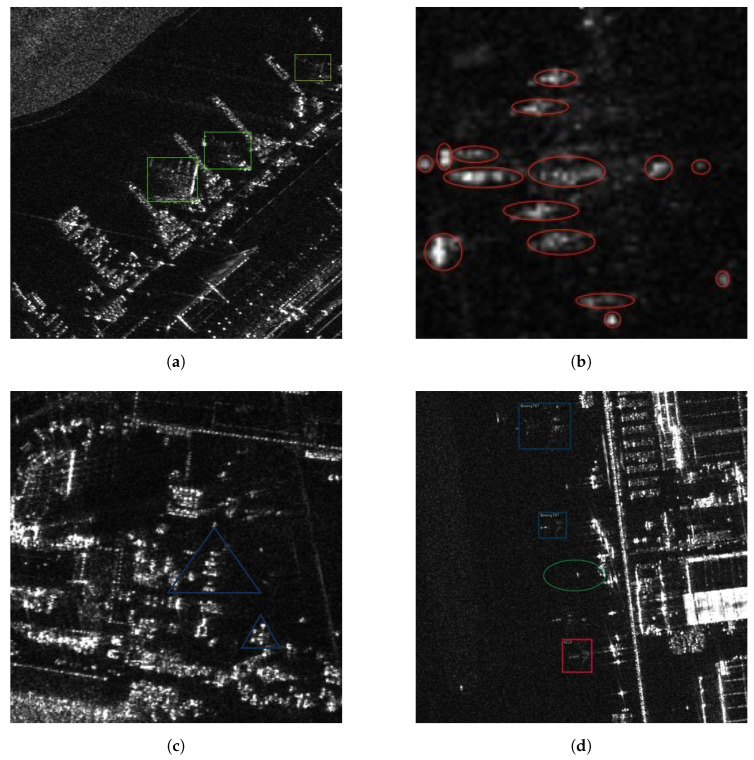
Aircraft and false objects in SAR images. (**a**) Aircraft objects. (**b**) Close-up of an aircraft. (**c**) False objects. (**d**) Incorrect annotations. Colored boxes represent real aircraft in the SAR images, and triangles represent false objects. The green ovals represent false targets incorrectly labeled as aircraft, and the small red ovals represent discrete features of the aircraft.

The unique imaging principles of SAR, coupled with the intricate structural features of aircraft, pose significant challenges to detection tasks. These challenges can be outlined as follows:

(1) **Discrete scattering features**. The particular imaging characteristics of SAR, combined with the intricate structure of aircraft, result in representations as scattered speckles rather than continuous forms, as shown in Figure 1a,b. This often leads to the segmentation of aircraft into disjointed components, undermining the integrity of detection results.

(2) **Interference from complex backgrounds**. Objects are susceptible to interference from surrounding backgrounds, such as terminal buildings and aprons, causing objects with similar scattering visual properties to be identified as aircraft objects, as shown in Figure 1c. This phenomenon brings about false alarms and missed detections.

(3) **Label errors**. If there are some label errors in the dataset, they might confuse neural networks and lead to unsatisfactory performance [17]. Some researchers have found that ImageNet [18], one of the most popular datasets in the world, has a non-negligible amount of noise in its annotations [19]. Considering that SAR object detection datasets are more challenging to annotate than optical images, it is reasonable to infer that SAR object detection datasets may contain more labeling errors. Figure 1d illustrates the issues of mislabeled and missing labels in the dataset.

To tackle the problems above, we proposed the scattering feature extraction and fusion network (SFEF-Net). The uniqueness of our approach stems from its comprehensive consideration of the distinctive characteristics of aircraft in SAR images and its targeted enhancements to achieve top-tier performance. First, we designed an innovative sparse convolution operator, which has a larger receptive field and more flexible sampling positions without increasing the number of parameters compared to traditional convolution. This characteristic plays a vital role in accurately extracting discrete aircraft features in SAR images. The receptive field of traditional convolution is a regular small rectangle because its essence is to calculate the weighted sum within a small rectangular range on the input feature map. In contrast, sparse convolution randomly selects a small number of positions within a larger neighborhood for the calculation of the output feature map. For example, nine positions are randomly sampled within a 5 × 5 neighborhood. Secondly, we developed the global information fusion and distribution module (GIFD) to combine feature maps across various levels and scales. To be specific, GIFD is composed of three components: feature alignment module (FAM), feature fusion module (FFM), and feature distribution module (FDM). The FAM takes the multi-scale features extracted by the backbone as input and aligns them to the same scale, which facilitates the processing by the FFM. The key component of FFM is the multi-head self-attention mechanism, which enables the flow of information across the feature map pyramid. The final FDM module completes the distribution of the feature maps. Therefore, GIFD has a global modeling capability, enabling the full fusion of feature maps and utilization of contextual information to enhance the accuracy of aircraft detection in complex backgrounds. Additionally, we introduced noise-robust loss to reduce the adverse effects of label noise. Incorrectly labeled samples, often manifested as outliers, are assigned lower weights, enabling the model to place greater emphasis on correctly labeled samples.

In summary, our main contributions can be outlined as follows:

(1) The SFEF-Net we proposed takes into account the discrete features of aircraft, complex background, and potential label noise in SAR images. Experimental results on the SAR-AIRcraft1.0 dataset [15] show that SFEF-Net outperforms existing methods.
(2) An innovative sparse convolution is proposed, which has a larger receptive field and more flexible sampling positions. Sparse convolution is more suitable for the extraction of discrete features.
(3) To improve accuracy in complex backgrounds, GIFD is utilized for feature fusion. GIFD has the capability of global modeling, enabling a comprehensive fusion of multi-scale feature maps and effective utilization of contextual information to suppress clutter.
(4) To mitigate the detrimental effects of label noise, we introduced a noise-robust loss that enables adaptive weight reduction for outliers.

The structure of this paper is outlined as follows. Section 2 reviews and summarizes the related work. Section 3 presents a detailed explanation of the three main components of the proposed approach. The content of Section 4 focuses on the experiments, covering the dataset description, evaluation criteria, and an analysis of the experimental results. Finally, Section 5 concludes this study with a concise summary. The findings of this study are expected to support further advancements in SAR-based aircraft detection research.

## 2. Related Work

### 2.1. Object Detection Algorithms Based on Deep Learning

In recent years, deep learning-based object detection algorithms have witnessed remarkable progress. These advancements, particularly those achieved through convolutional neural networks (CNNs), have led to notable improvements in the field. CNN-driven object detection methods are broadly divided into two types: anchor-based and anchor-free approaches. Anchor-based methods typically rely on bounding boxes with predetermined sizes and aspect ratios, while anchor-free methods predict object locations directly without predefined anchors. Furthermore, it is noteworthy that Transformer-based approaches have also begun to gain traction in the realm of object detection, injecting new vitality and innovation into the domain. These Transformer-based methods leverage the self-attention mechanism to improve detection accuracy and adaptability, especially when applied to large-scale datasets.

**Anchor-based detectors**. This type of detector divides the input image into multiple grids and assigns anchors, also known as priors or default boxes, to each grid. Anchors are predefined with specific sizes and aspect ratios. The model determines whether there are any interesting objects within each grid and calculates the offsets of these objects relative to the anchors. Anchor-based object detection can be further categorized into two-stage and one-stage methods.

A two-stage approach initially conducts a preliminary screening of predefined anchors, followed by further refinement through detailed classification and regression, which is how it derives its name. Notable examples of this methodology include the R-CNN series, such as R-CNN [20], Fast R-CNN [21], Faster R-CNN [22], and Cascade R-CNN [23]. These two-stage approaches are characterized by their high detection accuracy and have greatly contributed to the progress of the object detection domain.

In contrast, single-stage methods directly perform final classification and regression based on the anchors without the initial screening stage. As a result, these methods tend to be faster in terms of detection speed. Representative single-stage detectors comprise the YOLO series [24,25,26,27,28,29,30,31], SSD [32], and RetinaNet [33]. Due to their real-time performance, single-stage methods are widely applied in practical scenarios such as industrial settings.

**Anchor-free object detectors**. Instead, anchor-free detectors directly predict bounding boxes and confidence scores, eliminating the need for predefined anchor boxes. This method provides greater adaptability for detecting objects with varying sizes and aspect ratios. CornerNet [34], CenterNet [35], and FCOS [36] are representative works of anchor-free detectors.

CornerNet detects objects by predicting the positions of the top-left and bottom-right corners of bounding boxes and then grouping them to form the final detections. This method can effectively handle objects of varying sizes and shapes. CenterNet builds upon this idea by predicting object centers and the corresponding size of the bounding boxes, improving the robustness and accuracy of the detections. FCOS takes a different approach by predicting the four offsets from each point on a feature map to the four sides of the bounding box, thus enabling a fully convolutional end-to-end detection pipeline. Despite their advantages, anchor-free methods face challenges due to the absence of anchor boxes that provide prior knowledge about object locations and scales. This can result in a lower recall rate compared to anchor-based methods, as the network must learn to identify and localize objects from scratch without predefined references.

**Transformer-based detectors**. Leveraging the self-attention mechanism, the Transformer model has attained remarkable success across multiple domains, including natural language processing and computer vision [37,38,39,40]. For example, DETR [41], deformable DETR [42], and ViTDet [43] have introduced the Transformer into object detection from different perspectives and achieved high-quality performance. DETR formulated object detection as a direct set prediction problem and introduced a novel end-to-end approach using Transformers, removing the need for hand-designed components like anchor generation and non-maximum suppression. Building on the achievements of DETR, deformable DETR was developed to address the limitations of DETR’s fixed attention mechanisms. By introducing a deformable attention mechanism, it enables the model to focus on a sparse set of key sampling points around objects, thereby significantly enhancing efficiency and performance, particularly for high-resolution images. ViTDet applies the vision Transformer framework to object detection, demonstrating how pure Transformer models can achieve competitive results without convolutions. Research has shown that Transformer-based methods have an advantage on large-scale datasets but are prone to overfitting on small or medium-sized datasets. This is because Transformers, compared to convolutional methods, lack the inductive bias for images [44].

In summary, single-stage methodologies have successfully balanced speed and precision, making them well-suited for modest-scale datasets. Therefore, we have chosen single-stage approaches as our baseline. However, it should be noted that while deep learning algorithms have achieved significant success in natural image processing, their direct applications to SAR imagery are impeded by substantial disparities between the two modalities. Targeted improvements to existing methods are necessary to address these differences efficiently.

### 2.2. Multi-Scale Features Fusion for Object Detection

In general, the size of the objects of interest in a dataset varies greatly. Therefore, detecting multi-scale objects quickly and accurately is a fundamental problem in object detection. Leveraging multi-scale feature maps has been widely adopted as an effective solution to this challenge, as the CNN backbone network generates hierarchical feature maps. Specifically, the feature maps from shallower layers offer higher resolution and finer-grained details, making them particularly advantageous for detecting small objects. On the other hand, deep feature maps possess a broader receptive field and richer semantic information, making them well-suited for detecting large objects [45].

The groundbreaking design of the feature pyramid network (FPN) proposed in [46] introduced a structure that enables cross-level connections and information exchange. This design greatly enhances the detection performance of objects at multiple scales. Based on FPN, the path aggregation network (PANet) adds another bottom-up pathway, allowing for more comprehensive information fusion [47]. Similarly, EfficientDet introduces a weighted bi-directional feature pyramid network (BiFPN), which can dynamically learn the significance of every input feature map [48].

Nevertheless, the feature fusion networks built upon FPN suffer from inherent limitations in facilitating cross-layer information exchange. This is primarily due to their reliance on simplistic methods, such as addition or channel concatenation, for information flow between adjacent layers. Recognizing the potent global modeling capabilities offered by the multi-head self-attention (MHSA) mechanism, we have integrated MHSA into the feature fusion network, introducing a novel global information fusion and distribution (GIFD) module. This module enhances multi-scale feature fusion capabilities and achieves an optimal trade-off between speed and accuracy.

### 2.3. Classification Loss in Object Detection

In object detection, in addition to predicting the positions of the interested objects, we also need to predict their categories. The most commonly used loss function in category prediction is the cross-entropy (CE) loss [49]. The CE loss quantifies the difference between the predicted probability distribution and the ground truth. It encourages the predicted probabilities to be close to the ground truth probabilities, leading to accurate category predictions during object detection.

In [33], the authors argue that a significant limitation of early single-stage object detectors compared to two-stage detectors lies in the severe imbalance between foreground and background classifications. To address this, they proposed a novel focal loss, which dynamically adjusts the weight of easily classified background examples, thereby directing the model’s focus toward harder-to-classify foreground instances.

While both CE loss and focal loss demonstrate effective performance, it is crucial to note that their efficacy hinges on the dataset devoid of erroneous annotations. However, given the inherent challenges associated with annotating SAR object detection datasets, the presence of potentially inaccurate annotations is difficult to completely avoid, as it requires exceptionally proficient annotation experts. In response to this challenge, we introduce a novel noise-robust loss function capable of dynamically reducing the negative influence of outliers.

### 2.4. SAR Aircraft-Detection Methods with Deep Learning

Differing from traditional methods that require manual feature design [5,50,51,52,53,54,55], deep neural network-based methods can automatically learn feature extraction from the data and have higher performance and better generalization capabilities. Therefore, CNN-based aircraft detection for SAR images is currently a hot topic. He et al. [56] proposed a multi-layer parallel network with a component-based structure for detecting airplanes in SAR imagery. Experiments on TerraSAR-X imagery indicate the approach has a higher accuracy. Zhang et al. [57] introduced a scattering feature relation enhancement network, which can model the relationship between feature points. Zhao et al. [58] proposed a novel pyramid attention dilated network that improves the accuracy of SAR aircraft detection in complex backgrounds. Xiao et al. [59] designed an adaptive deformable network to fully utilize the strong scattering features of aircraft. Guo et al. [60] employed an aircraft detection framework built upon a feature pyramid network, integrating shallow high-resolution and deep high-semantic feature information. Wang et al. [61] proposed a multi-scale feature adaptive fusion module to assign learnable weights to each scale of the feature layers. Jia et al. [62] attempted to solve the islanding problem using multi-client joint training and model aggregation.

Previous methods have made significant contributions to aircraft detection in SAR images, but their approaches still have some issues. Firstly, aircraft in SAR images are discontinuous, and existing methods have not fully considered these characteristics, which can lead to the detection of incomplete results. Secondly, traditional FPN structures are unable to sufficiently exchange information across all feature layers, which limits the model’s ability to perform multi-scale detection in complex backgrounds. Thirdly, existing classification losses are overly sensitive to outliers and are greatly influenced by annotation errors. To address these issues, we have proposed the scattering feature extraction and fusion network (SFEF-Net), which has shown excellent performance.

## 3. Method

### 3.1. The Overall Structure of SFEF-Net

**Figure 2:**
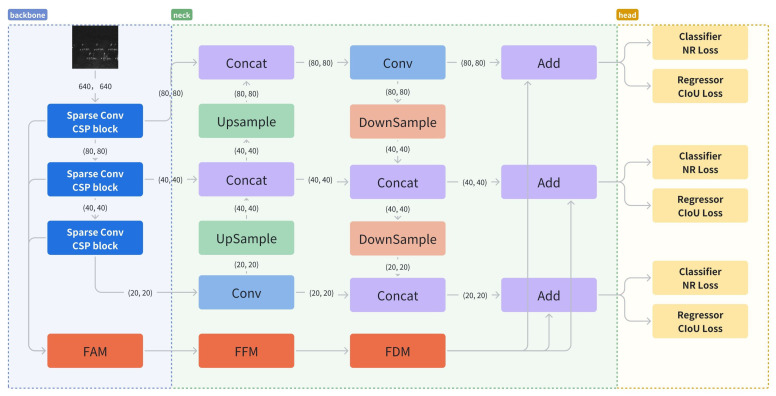
The overall structure of SFEF-Net is composed of three main sub-structures: backbone, neck, and head. Among them, the backbone integrated with sparse convolution extract features from the original input images, the neck, namely GIFD, fuses the features extracted by the backbone, and the head is responsible for predicting the positions and categories of potential objects. The NR Loss that we mentioned is used for category prediction.

As mentioned in Section 2.1, single-stage methods have shown good performance in terms of speed and accuracy. As one of the most outstanding algorithms in single-stage methods, YOLOv5 has been applied in many practical scenarios, and its generalization has been widely validated. Based on this, we have chosen YOLOv5 as our baseline. Figure 2 illustrates the overall structure of SFEF-Net, which consists of three components: backbone, neck, and head.

**Backbone.** The backbone processes the input images and generates multi-scale feature maps. Following the design principles of YOLOv5, we incorporated cross-stage partial connection [63] into the backbone because it has lightweight parameters but powerful feature extraction capabilities. In addition, we replaced some traditional convolutions in the backbone with the proposed sparse convolutions for extracting discrete features of aircraft.

**Neck.** The neck aggregates the feature maps extracted by the backbone, enhancing the model’s ability to detect objects across multiple scales. YOLOv5 uses PANet as its neck, which has inherent limitations in cross-layer information exchange. To address this drawback, we designed a novel global information fusion and distribution module that allows information to be exchanged between all feature maps, which improves the model’s detection accuracy in challenging background conditions.

**Head and Loss.** The head predicts the positions and categories of potential objects. To a certain degree, the value of the loss function reflects the quality of the prediction results, and the gradient of the loss function indicates the direction of parameter updates. For predicting positions, we utilize the CIoU loss, as described in [64]. In addition, to enhance category prediction, we developed a noise-robust loss function that mitigates the harmful effects of annotation noise.

### 3.2. Backbone: CSPDarkNet Integrated with Sparse Convolution

**Figure 3:**
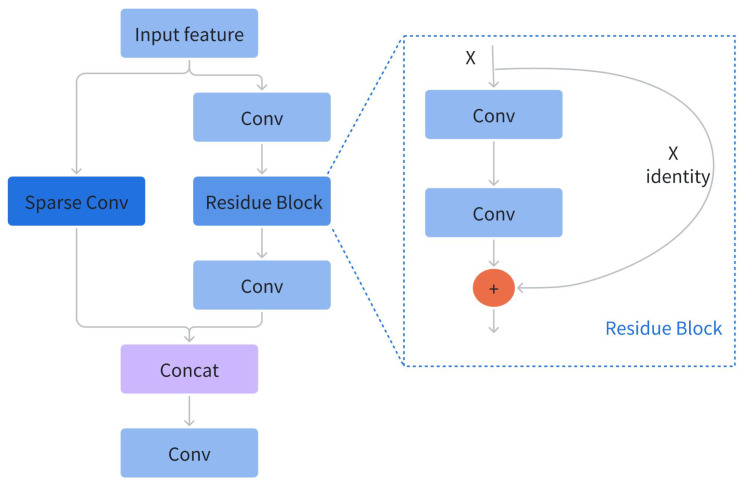
The CSP connection structure is composed of two branches: the right branch, built around a residue block as its main component, and the left branch, which incorporates sparse convolution.

**CSP connection.** As mentioned above, we use the CSPDarkNet integrated with sparse convolution as the backbone. As shown in Figure 3, the CSP connection forwards the input feature maps through two branches. The key feature of the right branch is the residual block, which was proposed in [11] to alleviate the issue of degradation in very deep neural networks. The left branch utilizes sparse convolution, whose detailed structure will be elaborated upon subsequently. CSP connection has powerful feature extraction capabilities while maintaining lightweight parameters. This is because traditional CNN architectures, such as DarkNet, adopt a single-branch design. This design leads to a large amount of redundant gradients during the forward propagation process, resulting in limited learning capacity. Instead, CSP employs two branches (left and right) that have different gradient flows. Particularly, the left branch does not have complex residual blocks, significantly lowering the amount of parameters and computational complexity. Moreover, the CSP connection has sufficient versatility, which allows it to be conveniently integrated into almost all existing CNN.

**Sparse Convolution.** The computation of 2D convolution involves two main steps: (1) selecting a subset of values from the input feature map *x* within a small, rectangular region *R*; (2) calculating a weighted aggregation of these selected points, where the weights are learnable parameters of the convolutional kernel. For example, for a 3 × 3 convolutional kernel, the sampling region $R_{TC}$ would be as follows:

(1) $$R_{TC}=\left\{\left(-1,-1\right),\left(-1,0\right),\left(-1,1\right),\left(0,-1\right),\left(0,0\right),\left(0,1\right),\left(1,-1\right),\left(1,0\right),\left(1,1\right)\right\}$$

where each element in R represents the vertical and horizontal offsets of the sampling points relative to the center of the convolutional kernel, respectively. Hence, for each point $p_{0}$ on the output feature map *y*, we have:

(2) $$y\left(p_{0}\right)=\sum_{p_{n}\in R}w\left(p_{n}\right)*x\left(p_{0}+p_{n}\right),$$

where $p_{0}=\left(i,j\right)$ denotes a spatial coordinate that iterates over all positions in the output feature map *y*. $y(p_{0})$ represents the activation value at coordinate $p_{0}$ in the output feature map *y*. The set *R* refers to the collection of sampling offsets relative to the kernel’s center; for the standard 3 × 3 traditional convolution shown, $R=R_{TC}$ as defined in Equation (1). Each $p_{n}=\left(\Delta i,\Delta j\right)$ within *R* is a specific sampling offset. $w(p_{n})$ is the learnable kernel weight associated with the offset $p_{n}$. Finally, $x(p_{0}+p_{n})$ represents the activation value from the corresponding location in the input feature map *x*, accessed by adding the offset $p_{n}$ to the output coordinate $p_{0}$.

Dilated convolution [65], also known as atrous convolution, is an extension of traditional convolution. It selects sampling points over a larger range. For example, with a dilation rate of 1 and a kernel size of 3, the sampling range $R_{DC}$ will be:

(3) $$R_{DC}=\left\{\left(-2,-2\right),\left(-2,0\right),\left(-2,2\right),\left(0,-2\right),\left(0,0\right),\left(0,2\right),\left(2,-2\right),\left(2,0\right),\left(2,2\right)\right\}$$

Dilated convolution offers a larger receptive field in comparison to traditional convolution, making it more suitable for extracting discrete aircraft features in SAR images. Several studies, such as [57,58], have successfully utilized dilated convolution to achieve certain progress. However, dilated convolution still samples at fixed positions, which results in inherent information loss. The fundamental reason for information loss is that different positions on the input feature map are sampled with entirely different probabilities. If a point $p\in R_{DC}$, then its sampling probability is 1; otherwise, it is 0. If the crucial discrete features of the airplane happen to be located outside of $R_{DC}$, there is a high possibility that the detection results will be incorrect.

To overcome the limitations of fixed sampling in dilated convolution, the sparse convolution we proposed further expands on this concept by introducing channel-specific randomized sampling patterns. The fundamental computation remains a weighted aggregation similar to Equation (2), but the crucial difference lies in how the sampling offsets are determined.

Let $y_{c}\left(p_{0}\right)$ denote the activation at position $p_{0}$ in the *c*-th output feature map (where $c\in \{1,\ldots ,C_{out}\}$), and $x_{c_{in}}\left(p_{0}\right)$ denote the activation at position $p_{0}$ in the $c_{in}$-th input feature map (where $c_{in}\in \left\{1,\ldots ,C_{in}\right\}$). The sparse convolution operation is defined as:

(4) $$y_{c}\left(p_{0}\right)=\sum_{c_{in}=1}^{C_{in}}\sum_{p_{n}\in R_{\mathrm{SC}}\left(c\right)}w_{c,c_{in}}\left(p_{n}\right)\cdot x_{c_{in}}\left(p_{0}+p_{n}\right)$$

where $w_{c,c_{in}}\left(p_{n}\right)$ represents the learnable kernel weight and $R_{SC}\left(c\right)$ is the channel-specific set of *K* randomly sampled offsets for the *c*-th output channel.

The generation and properties of the sampling offset set $R_{SC}\left(c\right)$ are crucial:

- **Initialization and Fixation:** The set $R_{SC}\left(c\right)$ for each output channel *c* is generated **once during model initialization**. Specifically, for each *c*, K=9 sampling offsets $p_{n}=\left(\Delta i,\Delta j\right)$ are selected **uniformly at random without replacement** from a predefined 5×5 neighborhood (W=5) centered at the origin. These randomly determined sampling positions **remain fixed** throughout all subsequent training and inference stages.
- **Channel Independence:** Crucially, the sampling patterns $R_{SC}\left(c\right)$ are generated **independently** for each output channel *c*. This means $R_{SC}\left(c_{1}\right)$ is generally different from $R_{SC}\left(c_{2}\right)$ if $c_{1}\neq c_{2}$.

This per-channel independent random sampling strategy allows different channels to potentially focus on different spatial locations, leading to enhanced coverage. Consider a specific feature point $p^{\prime }$ within the 5×5 sampling neighborhood. The probability of it being selected by a single channel is $p=K/W^{2}=9/25=0.36$. The probability of this point *not* being selected by *any* of the $C_{out}$ independent output channels is ${\left(1-p\right)}^{C_{out}}$. As $C_{out}$ increases, this probability rapidly approaches zero (e.g., for $C_{out}=32$, it is $\approx 1.9\times 10^{-7}$), ensuring high coverage of the input feature map and mitigating potential information loss. It is important to note that while the randomly generated sampling locations $R_{SC}\left(c\right)$ remain fixed after initialization, the associated kernel weights *w*, initialized using standard methods and subsequently optimized via backpropagation during training just like conventional convolutional layers. Sparse convolution, as implemented here, does not involve learning or dynamically adjusting the sampling locations themselves.

**Figure 4:**
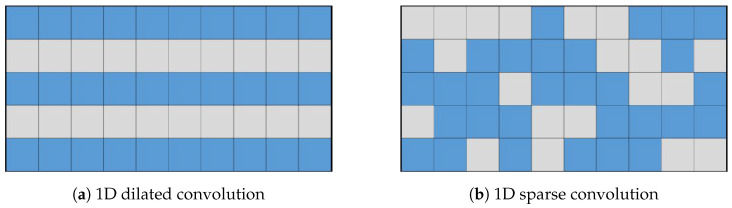
Illustration comparing sampling patterns of 1D dilated convolution and our proposed sparse convolution over multiple output channels. Each grid represents the sampling positions relative to the output location across different output channels (columns). The vertical axis (5 rows) conceptually represents a 1D input neighborhood (e.g., corresponding to a receptive field or sampling range of size 5). Blue cells indicate positions sampled by the convolution kernel for that specific output channel, while gray cells represent positions within the neighborhood that are *not* sampled by that channel. (**a**) Dilated convolution uses a fixed, identical sampling pattern (blue rows are the same) across all output channels, potentially missing information in the discarded (gray) positions consistently. (**b**) Sparse convolution employs independent, random sampling patterns for each output channel (blue cells vary across columns). This allows different channels to cover different input positions, significantly increasing the probability that any given input position is sampled by at least one channel, thus mitigating information loss.

To illustrate the fundamental difference in sampling strategy and its impact on information coverage more clearly, let us consider a simplified one-dimensional analogy, as depicted in Figure 4. While our actual application involves 2D convolutions, this 1D example effectively highlights the core distinction between fixed versus randomized channel-independent sampling. Let the input feature map be $x\in R^{5}=\left\{x_{1},x_{2},x_{3},x_{4},x_{5}\right\}$. The output channel of the dilated convolution $C_{d}$ is 10, with a kernel size of 3 and a dilation rate of 1. The parameters of the sparse convolution $C_{s}$ are kept consistent with the dilation convolution. The dilation convolution and sparse convolution are depicted in Figure 4, with each column representing an output channel. The blue cells represent the sampled positions, while the gray cells represent the discarded positions. In the dilation convolution, all channels discard $x_{2}$ and $x_{4}$. If important features happen to be present in these positions, it can result in inherent information loss. On the other hand, in sparse convolution, similar information loss does not occur.

Although presented in 1D for simplicity, the same principle applies to 2D sparse convolution: by having each output channel independently sample random 2D offsets $p_{n}\in R_{SC}\left(c\right)$ within a larger neighborhood, we ensure diverse coverage of the 2D input feature map across the channel dimension.

In addition, it is worth noting that sparse convolution does not increase the number of parameters compared to traditional and dilated convolution. When the kernel size of the convolution is *k*, and the input and output channels are $C_{in}$ and $C_{out}$, respectively, the number of parameters for all three of them is $C_{out}*C_{in}*k^{2}$. To provide a clearer comparison, we have summarized the main characteristics and illustrations of the three methods in Table 1.

### 3.3. Global Information Fusion and Distribution (GIFD) Module

**Figure 5:**
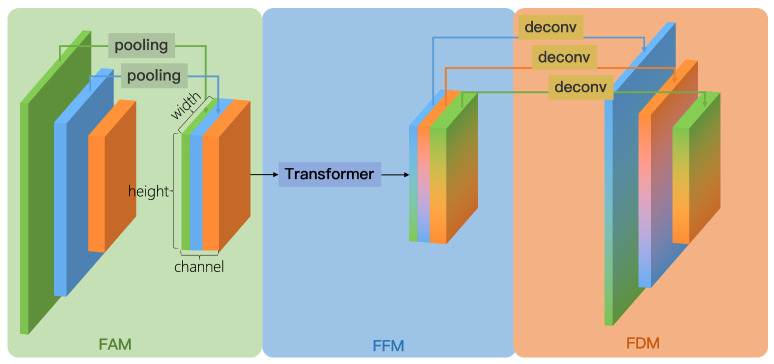
Illustration of the global information fusion and distribution (GIFD) module. (**left**) The feature alignment module (FAM) takes multi-scale inputs (P3-green, P4-blue, P5-orange) and aligns them to the P5 scale using downsampling operations, followed by channel concatenation. (**center**) The feature fusion module (FFM) processes the aligned features using Transformer encoders. (**right**) The feature distribution module (FDM) splits the fused features and employs upsampling operations to distribute information back to the original P3, P4, and P5 scales for the detection heads.

We designed the global information fusion and distribution (GIFD) module to effectively fuse multi-scale feature maps extracted by the backbone (typically P3, P4, and P5, corresponding to stride 8, 16, and 32, respectively), enhancing the model’s multi-scale detection capability while achieving a balance between speed and accuracy. The conceptual illustration of GIFD is shown in Figure 5. Unlike traditional feature fusion networks like FPN and PANet, which primarily exchange information between adjacent layers and can suffer from information loss across distant scales, GIFD employs a global fusion mechanism via its feature fusion module (FFM). GIFD consists of three key sub-modules: feature alignment module (FAM), feature fusion module (FFM), and feature distribution module (FDM).

**Feature Alignment Module (FAM).** As depicted conceptually in Figure 5 (left part), the FAM receives multi-scale feature maps P3, P4, and P5 from the backbone (corresponding to strides 8, 16, and 32, respectively). To prepare these features for efficient global fusion in the subsequent FFM module, FAM aligns them all to a common, reduced spatial resolution, specifically, the **P5 scale** (stride 32). This spatial alignment is achieved by applying appropriate downsampling operations to the higher-resolution feature maps P3 and P4 to match the spatial dimensions of P5, for example, using average pooling or max pooling. The specific choice of downsampling method is not considered a critical design element here, as the primary goal is simply to reduce the spatial resolution efficiently to achieve scale alignment before concatenation. Following these alignment steps, the processed P3, P4, and P5 feature maps, now all sharing the same P5 spatial resolution, are concatenated along the channel dimension. This produces the unified feature map $F_{align}$, which compactly represents information from all input scales at the minimal resolution, thus reducing the computational load for the FFM while maintaining low latency.

**Figure 6:**
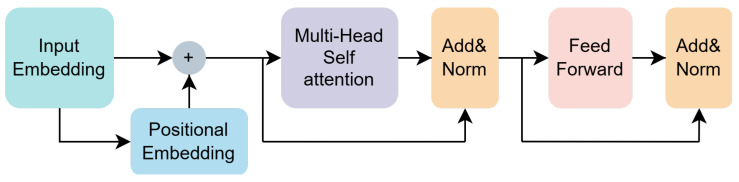
The architecture of the Transformer encoder, whose key component is multi-head self-attention. Self-attention possesses the capability for global modeling, and we have utilized this characteristic to enhance the model’s multi-scale detection ability. Following the multi-head attention, the architecture includes normalization and feed-forward layers, along with the incorporation of residual connections.

**Feature Fusion Module (FFM).** Inspired by ViT [39], the FFM takes the concatenated and aligned feature map $F_{align}$ (at P5 scale) as input. It employs Transformer encoders, whose key component is multi-head self-attention (MHSA), to perform global information fusion across all spatial locations and aggregated channel information. The detailed architecture of the Transformer encoder used is shown in Figure 6. By leveraging the Transformer’s ability to model long-range dependencies, FFM effectively breaks the information flow barriers inherent in traditional convolutional neck structures. The output of the FFM is the fused feature map $F_{fuse}$, which maintains the same P5 spatial resolution and channel dimension as $F_{align}$.

The architecture of the Transformer encoder is shown in Figure 6. Generally, the Transformer encoder takes a sequence of 1D vectors as input and outputs vectors of the same length. To handle 2D images, we reshape the feature map $F_{align}\in R^{H\times W\times C}$ into ${\tilde{F}}_{align}\in R^{N\times C}$, where *H*, *W*, and *C* represent the height, width, and channels of $F_{align}$, respectively, and N=HW represents the number of feature points. In addition, we employ a learnable linear layer as positional embedding to capture the positional information in the sequence, allowing the model to better learn the relationships between elements at different positions in the input sequence. Therefore, the input vector sequence, after positional encoding, goes through the MHSA layer, normalization layer, and feed-forward layer, resulting in the fused feature map $F_{fuse}$.

**Feature Distribution Module (FDM).** The fused feature map $F_{fuse}$ obtained from FFM, while contextually rich, exists only at the single, reduced P5 scale. To generate the multi-scale feature representations required by standard object detection heads, the FDM module distributes this fused information back to multiple spatial resolutions, specifically targeting the original P3, P4, and P5 scales. As illustrated conceptually in Figure 5 (right side), FDM effectively performs the inverse process of FAM. First, the channels of $F_{fuse}$ are split into three segments, corresponding to the target P3, P4, and P5 output paths. The segment destined for the P5 output path passes through a 3 × 3 convolutional layer, with stride 1 and padding 1. This operation maintains the P5 spatial resolution while potentially refining the features, producing the final $P5_{out}$ feature map. The segment allocated for the P4 output path is first upsampled by a factor of 2 to restore the P4 spatial resolution (corresponding to stride 16). This upsampling can be achieved using standard methods, for example, a transposed convolution layer ultimately producing the $P4_{out}$ feature map. Likewise, the segment corresponding to the P3 output path undergoes a similar process but with an upsampling factor of 4 to restore the P3 spatial resolution (stride 8), potentially also involving methods like transposed convolution, which yields the final $P3_{out}$ feature map. The resulting set of feature maps $P3_{out},P4_{out},P5_{out}$, possessing spatial resolutions corresponding to strides 8, 16, and 32, respectively, and appropriate channel dimensions, directly meet the requirements for the subsequent multi-scale detection heads. This completes the process of the global information fusion and distribution (GIFD) module.

### 3.4. Noise-Robust Loss

To mitigate the negative impact of incorrect annotations in the dataset on the prediction results, we have designed a noise-robust loss. The NR Loss assigns dynamic weights to erroneous annotations, which are often outliers during the training process, allowing the model to focus more effectively on the correct annotations.

Our NR Loss is an improvement upon the classical cross-entropy loss function. The formula for binary cross-entropy (BCE) is as follows:

(5) $$BCE\left(y,\hat{y}\right)=-y\log\left(\hat{y}\right)+\left(1-y\right)\log\left(1-\hat{y}\right)$$

where *y* and $\hat{y}$ represent ground truth and prediction probability, respectively. Cross-entropy measures the difference between the predicted probabilities of the model and the ground truth. By minimizing the cross-entropy loss, the model can better fit the training data and improve its classification performance. However, one main drawback of cross entropy is that it is too sensitive to outliers [66]. For example, when the ground truth is 0 and the model’s predicted result varies from 0 to 1, the value of the cross-entropy loss function will change. On the one hand, when the predicted probability is close to 0, the loss grows slowly. On the other hand, when the predicted probability approaches 1, the loss escalates rapidly. Formally, NR Loss adds a dynamic adjustment factor to the cross-entropy loss. We define NR Loss as follows:

(6) $$NR\left(y,\hat{y}\right)=\left[1-\exp\left(\frac{|y-\hat{y}|-1}{\alpha }\right)\right]BCE\left(y,\hat{y}\right)$$

where α is a hyperparameter that represents the degree of penalty for outliers. In NR Loss, we use $|y-\hat{y}|$ to measure the likelihood of a sample being an outlier. When $|y-\hat{y}|$ is close to 1, it indicates a significant discrepancy between the model’s prediction and the ground truth, suggesting that it is likely an outlier and will be assigned a lower weight. On the other hand, when the model’s prediction is similar to the ground truth, it suggests that the sample is less likely to be an outlier and will receive a relatively larger weight. We can also adjust the degree of penalty for outliers by changing α. When α increases, the penalty becomes stronger, and vice versa.

## 4. Experiments

### 4.1. Dataset

**Figure 7:**
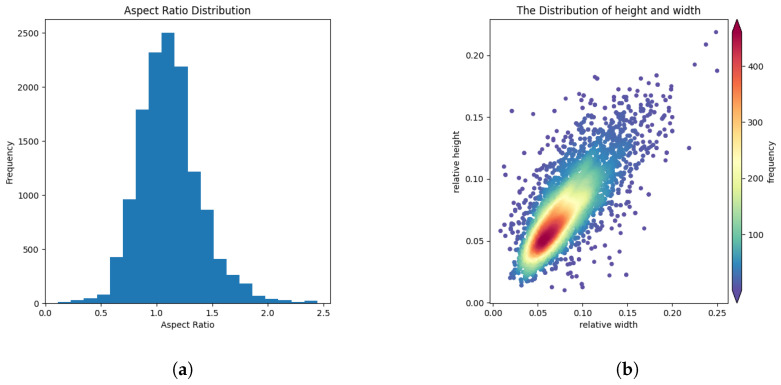
The variability in the aspect ratios of bounding boxes in SAR-AIRcraft-1.0. The aspect ratio of the aircraft is distributed around 1, and the size of the aircraft varies significantly. (**a**) The variability in the aspect ratios of bounding boxes. (**b**) Distribution of relative size variations in bounding boxes.

Currently, there is only one publicly available dataset for multi-class SAR image aircraft detection, which is SAR-AIRcraft-1.0 [15]. As a result, we use this dataset to validate our method. All the images in the SAR-AIRcraft-1.0 dataset were acquired from the Gaofen-3 satellite with single polarization, 1 m spatial resolution, and spotlight imaging. SAR-Aircraft-1.0 consists of 4368 images and 16,463 aircraft target instances. The aspect ratio and relative scale distribution of these instances are shown in Figure 7. From the graph, it can be observed that the aspect ratio is predominantly centered around 1, and the bounding boxes mainly consist of small to medium-sized objects.

**Figure 8:**
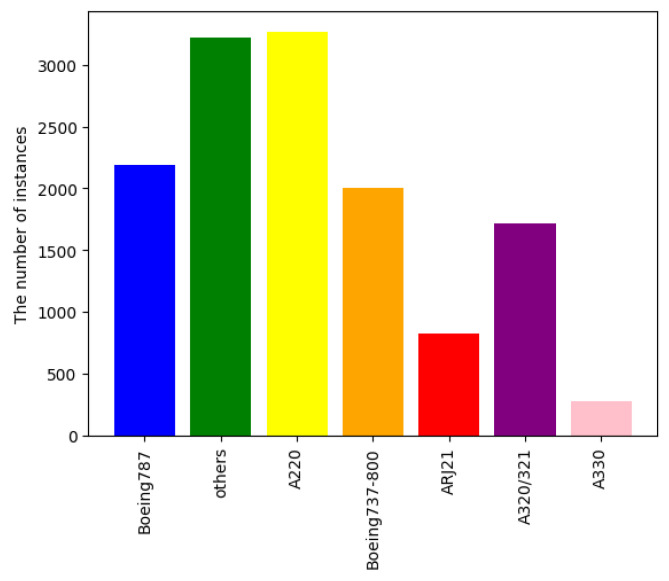
The distribution of instances across categories, including Boeing 787, A220, A330, and so on. It can be observed that there is an imbalance in the instances of different categories.

In the SAR-Aircraft-1.0 dataset, seven categories of aircraft have been annotated, including models such as Boeing 787 (Seattle, WA, USA), Airbus A220, and A330 (Toulouse, France).The dataset provides a comprehensive annotation for each category, capturing various angles and conditions under which the aircraft images were taken. Figure 8 illustrates the number of instances in each category, highlighting a noticeable imbalance among them. For instance, some categories may contain significantly more instances than others, which could potentially impact the performance of machine learning models trained on this dataset. This imbalance necessitates consideration in the model training process to ensure fair and accurate detection across all aircraft categories.

### 4.2. Experimental Setup

The proposed SFEF-Net was trained on an NVIDIA RTX 3090 GPU. We employed data augmentation techniques such as random rotation and random flip. The software version and other experimental hyperparameters are detailed in Table 2. To ensure a fair comparison in our experiments, the key baseline models, namely YOLOv5 [28], were trained and evaluated under identical conditions as our SFEF-Net. This includes using the same input resolution (640 × 640), the same number of training epochs, and the same data augmentation techniques.

### 4.3. Evaluation Metrics

We use precision and recall as evaluation criteria, and their formulas are as follows:

(7) $$\mathrm{Precision}=\frac{TP}{TP+FP}$$

(8) $$\mathrm{Recall}=\frac{TP}{TP+FN}$$

where TP, FP, and FN represent the number of true positives, false positives, and false negatives samples, respectively. Precision emphasizes the reliability of positive predictions, while recall measures the model’s ability to find all positive samples. However, precision and recall are typically trade-offs, meaning that when one increases, the other tends to decrease. So, we also use the F1-score, a metric that takes into account both precision and recall in a balanced way. The formula of the F1-score is as follows:

(9) $$F1=\frac{2\times \mathrm{Precision}\times \mathrm{Recall}}{\mathrm{Precision}+\mathrm{Recall}}$$

Additionally, we also employed the AP50 and AP50-95 evaluation metrics, which are widely used in the Microsoft Common Objects in Context dataset [67]. This is because precision, recall, and F1-score are all related to the classification confidence threshold, while average precision (AP) considers the overall performance of the model at different thresholds. The AP is as follows:

(10) $$\mathrm{AP}=\int_{0}^{1}p(r)dr$$

AP50 represents the AP when the IoU threshold is set to 0.5, while AP50-95 represents the mean AP when the IoU threshold ranges from 0.5 to 0.9 with an increment of 0.05.

### 4.4. Experimental Results

To validate the effectiveness of the proposed method, we compared it with several popular object detection algorithms, including Faster R-CNN, RetinaNet, SSD, YOLOv5, and others. Table 3 presents the performance of these methods. Compared to existing algorithms, the proposed SFEF-Net has achieved top-level performance on multiple evaluation metrics, especially AP50-95. This indicates that our algorithm can achieve good results at different IoU thresholds. In other words, our method has sufficient robustness to handle various complex situations in practical scenarios.

Furthermore, to provide a comprehensive assessment of the practical applicability of SFEF-Net, we analyzed its computational complexity and inference efficiency in comparison to the baseline methods, as detailed in Table 4. This table presents the number of parameters (Params), theoretical computational load (GFLOPs at 640 × 640 input), and practical inference speed (FPS measured on an NVIDIA RTX 3090 with FP16 precision and batch size 1). It can be observed that our proposed SFEF-Net, with 8.3M parameters and 18.9 GFLOPs, exhibits a modest increase in complexity compared to YOLOv5s (7.2M Params, 16.4 GFLOPs) but remains significantly lighter than methods like Faster R-CNN (41.8M Params, 180.5 GFLOPs) or Cascade R-CNN (69.2M Params, 260.3 GFLOPs). In terms of inference speed, SFEF-Net achieves 202.8 FPS, which is highly competitive and comparable to YOLOv8s (191.0 FPS) and slightly slower than YOLOv5s (242.1 FPS) under identical conditions. Considering the substantial improvement in detection accuracy (AP50-95 of 65.2% vs. 59.1% for YOLOv5s and 59.8% for YOLOv8s, as shown in Table 3), the slight increase in computational cost and comparable high inference speed demonstrate a favorable trade-off. This balance between high accuracy and efficient processing underscores the potential of SFEF-Net for practical deployment in demanding SAR image analysis tasks.

The superior performance of SFEF-Net can be attributed to several key factors. Firstly, SFEF-Net leverages sparse convolution with larger and more flexible receptive fields, which allows it to effectively capture discrete aircraft features in SAR images. This approach surpasses the limitations of traditional convolutions and dilated convolutions by providing a more adaptable sampling mechanism. Sparse convolution enables the network to focus on critical regions of interest, thereby enhancing feature extraction and improving detection accuracy.

Secondly, the GIFD module plays a crucial role in enhancing the model’s performance. Unlike conventional feature fusion methods that only allow information exchange between adjacent layers, GIFD facilitates global information effectively flowing across all layers. This comprehensive fusion capability ensures that features at different scales are effectively integrated, enhancing the model’s capacity to detect objects with varying sizes and shapes in SAR images. This enhancement is particularly beneficial for multi-scale detection capabilities, especially in complex backgrounds, where traditional methods often struggle to maintain accuracy and robustness.

Moreover, the incorporation of the NR-Loss is instrumental in boosting the robustness of SFEF-Net. NR-Loss specifically targets and mitigates the negative impact of outliers, which are prevalent in SAR images due to noise and other distortions. By reducing the influence of these outliers, NR-Loss helps maintain high detection accuracy even in challenging conditions.

Overall, SFEF-Net is designed with the unique characteristics of SAR image-based aircraft detection in mind. The algorithm takes into account the high variability in aircraft appearances and the complex backgrounds often present in SAR images. This consideration is reflected in the network architecture and the training process, resulting in a model that is not only accurate but also highly adaptable to real-world scenarios. The combination of sparse convolution, GIFD, and NR-Loss, along with a design tailored to the specific challenges of SAR image aircraft detection, enables SFEF-Net to achieve outstanding performance. These innovations collectively enhance the model’s feature extraction, information fusion, and robustness, making it a powerful tool for accurate and reliable object detection in complex environments.

**Figure 9:**
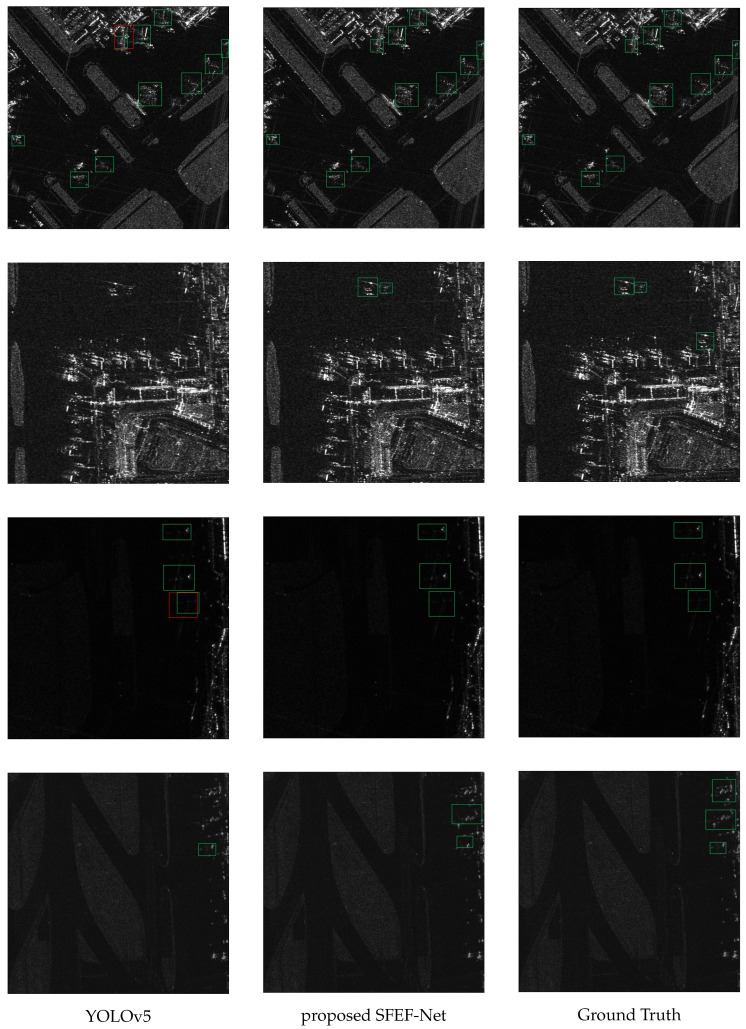
The comparison of the detection results between the baseline and the proposed SFEF-NET. The first two columns represent the detection results of YOLOv5 and the proposed SFEF-Net, respectively, while the last column represents the ground truth. In Ground Truth, all annotation boxes are marked in green. In the model’s detection results, correct detection boxes are marked in green, and false alarms are marked in red. It is evident that the detection results of YOLOv5 exhibit some missed and false objects, whereas our method shows a significant improvement.

Figure 9 shows the detection result contrasts among the baseline, our approach, and the ground truth. The left and middle columns display the detection results of the baseline and our method, respectively, while the right column shows the ground truth. Analyzing the three sets of results, it becomes apparent that the baseline method suffers from several missed detections and false positives. These issues are particularly prominent in situations where distinguishing discrete aircraft components from the surrounding background is difficult.

In contrast, our method demonstrates significantly better detection performance. Specifically, there is a notable reduction in both the rates of missed and false detections. The visual comparison indicates that our method’s detections closely align with the ground truth, demonstrating its high precision and reliability. This improvement is evident across various complex scenarios depicted in the images, confirming the robustness and effectiveness of our approach.

The ground truth column serves as a benchmark, clearly illustrating the areas where the baseline method falls short. Our method’s results show fewer errors, reflecting its enhanced capability to accurately detect aircraft in SAR images. This alignment with the ground truth underscores the practical applicability of our method in real-world detection tasks, where accuracy and reliability are paramount.

In summary, the visual comparison in Figure 9 showcases the substantial advancements our method offers over the baseline, particularly in handling complex detection scenarios. These results validate the effectiveness of the proposed enhancements, demonstrating their significant impact on improving detection accuracy in SAR image-based aircraft detection.

### 4.5. Ablation Studies

To additionally verify the efficiency of our designed SFEF-Net, we performed a series of ablation experiments, meticulously examining various components of our model. These experiments employed YOLOv5 and YOLOv8 as the baseline models to provide a robust comparison framework.

The first set of experiments focused on the impact of incorporating sparse convolution into the feature extraction network. Sparse convolution is designed to operate with flexible sampling positions, allowing for a more nuanced extraction of features, particularly in scenarios with complex patterns like aircraft scattering. Table 5 presents the comparative results of using sparse convolution versus traditional convolution on both YOLOv5 and YOLOv8. The results clearly demonstrate that integrating sparse convolution leads to a marked improvement in detection precision. Specifically, the precision metric increased by 1.6% when sparse convolution was utilized, and a similar improvement was observed in YOLOv8. This consistent enhancement underscores the ability of sparse convolution to better capture and highlight critical features within the objects, regardless of the underlying model architecture.

**Figure 10:**
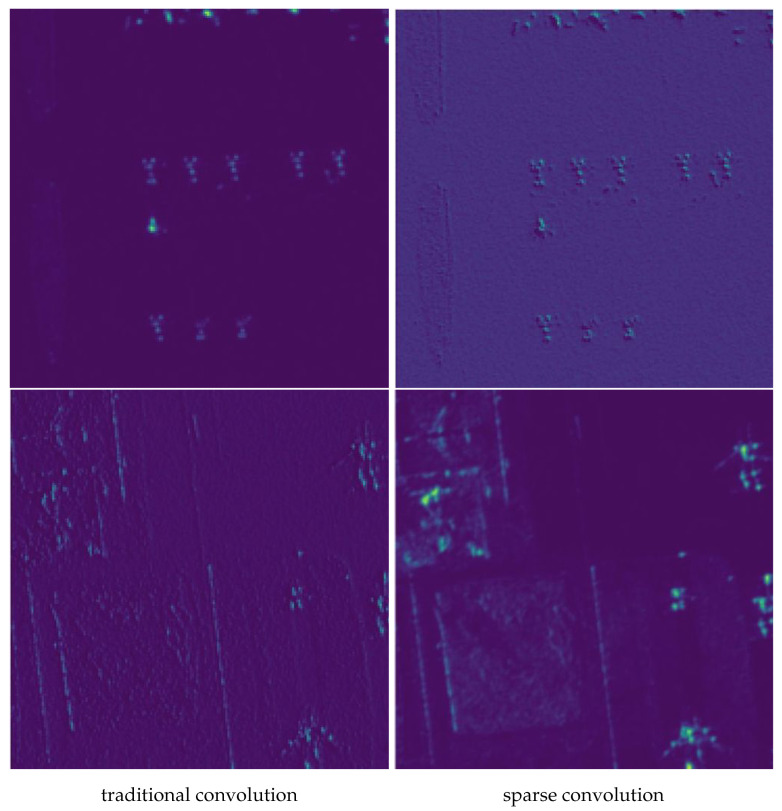
Comparison of visualization of feature maps for traditional convolution and sparse convolution.

To provide a more tangible illustration of these improvements, we visualized the feature maps generated by both traditional and sparse convolution methods. Figure 10 showcases these visualizations, with the top row representing the feature maps produced by traditional convolution, and the bottom row displaying those from sparse convolution. The visual comparison reveals that sparse convolution produces feature maps with a significantly enhanced focus on key regions of the aircraft, such as the edges and distinct structural elements. This enhanced focus is indicative of sparse convolution’s superior capability in feature extraction, which translates to higher precision in object detection tasks. Recognizing the clear benefits of sparse convolution, we adopted it in all subsequent ablation experiments, particularly those evaluating the GIFD and NR Loss function.

Secondly, we investigate the effect of different feature fusion networks on the detection results. In this ablation experiment, we compare three different feature fusion networks, namely FPN [46], PANet [47], and our proposed GIFD, and their comparisons are shown in Table 6. Based on the evaluation metrics presented in the table, it is evident that GIFD exhibits a significant improvement in AP50-95. AP50-95 comprehensively considers the detection accuracy under different classification confidence thresholds and regression intersection over union thresholds. This indicates that our GIFD has higher localization accuracy and stronger robustness. This is due to GIFD overcoming the inherent information exchange loss in traditional feature fusion networks. By introducing a self-attention mechanism, GIFD possesses long-range modeling capabilities, which enhances the model’s effectiveness in handling complex scenes.

Finally, regarding the study on NR Loss, we conducted two experiments. The first experiment used the original dataset, which contained all the annotation data. The second experiment intentionally removed a certain proportion of the annotated data to simulate missing annotations.

In Table 7, a comparison was made between the use of NR Loss under the two aforementioned settings. It is evident that regardless of whether NR Loss is used, there is a significant decrease in detection performance when a certain proportion of annotations are missing, as expected. This is because the missing annotations for aircraft that should have been in the foreground are erroneously labeled as background, causing confusion for the model.

After using NR Loss, the mislabeled outliers are assigned smaller weights, which makes the model pay more attention to the correctly labeled samples, thereby significantly improving the recall rate.

### 4.6. Feature Visualization

**Figure 11:**
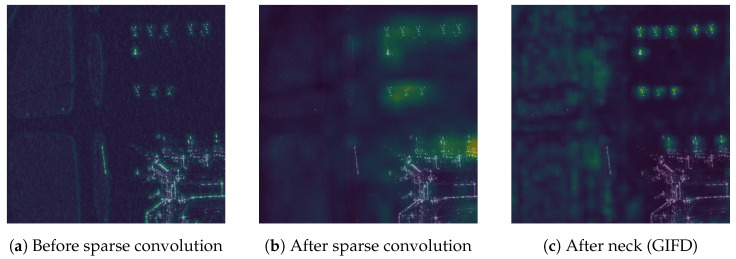
Visualization of feature maps at different stages of the proposed SFEF-Net. (**a**) Feature map before a sparse convolution block. (**b**) Feature map after the sparse convolution block. (**c**) Feature map after processing by the neck (GIFD module). Brighter colors indicate higher activation values, showing feature refinement and target focus progression.

To gain qualitative insights into how SFEF-Net processes information and the roles of its key components, we visualize the feature maps at different stages of the network using representative SAR images. Figure 11 illustrates the evolution of features for an example input image. We compute the channel-wise average of the feature maps at each selected stage and display them as heatmaps.

Figure 11a shows the feature map before entering our proposed sparse convolution blocks. While some activations corresponding to potential aircraft targets (top-right) and background structures (bottom-right) are visible, the overall feature representation is relatively diffuse.

Figure 11b displays the feature map immediately after the sparse convolution block. Compared to Figure 11a, we observe a noticeable enhancement and concentration of activations on the discrete scattering points associated with the aircraft targets. The intensity in these regions is significantly higher, suggesting that the sparse convolution effectively captures and emphasizes these critical sparse features, which aligns with our design motivation. Concurrently, some background clutter appears relatively suppressed.

Figure 11c presents the feature map after processing by the neck, which incorporates our GIFD module. This stage demonstrates further refinement. The activations for the aircraft targets are further sharpened and localized, likely benefiting from the multi-scale feature fusion and global context modeling capabilities of the GIFD module. Importantly, surrounding background regions show considerably lower activation compared to the target areas, indicating effective background suppression facilitated by the neck structure.

Overall, these visualizations provide qualitative evidence supporting the effectiveness of our proposed components. The sparse convolution enhances the response to discrete SAR features, and the GIFD module effectively fuses multi-scale information while suppressing background interference, ultimately leading to a feature representation well-suited for accurate aircraft detection.

## 5. Conclusions

In this study, we propose a new method for aircraft detection in SAR imagery: SFEF-Net, which mainly consists of three innovations. Firstly, we propose a novel sparse convolution for extracting discrete aircraft features. Compared to traditional convolution, sparse convolution samples a small number of points in a larger neighborhood. Therefore, without increasing computational and parameter requirements, sparse convolution has a larger and more flexible receptive field, making it suitable for extracting discrete features. Secondly, our proposed global information fusion and distribution (GIFD) module uses self-attention methods for global modeling and fusion of deep and shallow feature maps. GIFD consists of three sub-modules: feature alignment module (FAM), feature fusion module (FFM), and feature distribution module (FDM), enhancing the network’s multi-scale feature fusion capability and achieving a balance between speed and accuracy. Lastly, we introduce a noise-robust (NR) loss to alleviate the negative impact of mislabeling in the dataset on detection results. The NR Loss assigns smaller weights to outliers, enabling the model to focus more on correctly labeled instances. Extensive experiments on the SAR Aircraft 1.0 dataset demonstrate that SFEF-Net surpasses existing aircraft detection methods, achieving state-of-the-art performance.

Although our method could achieve good detection results for aircraft in SAR images, there are still some limitations. For example, the current publicly available dataset is relatively small, and the annotated categories are not very diverse. This limits the complexity of neural network models and can lead to overfitting. A current research trend is towards larger datasets and more complex models, such as models represented by Transformer. With the increasing capability to acquire SAR data, we look forward to larger datasets in the future to improve the robustness and credibility of detection results.

Looking ahead, one promising direction for future research involves integrating auxiliary information sources, building upon the foundation of enhancing detection from intrinsic SAR features laid in this work. Fusing SAR data with complementary modalities, such as optical imagery, could potentially provide richer contextual information and significantly improve detection performance and robustness. However, effectively addressing the associated challenges in data co-registration and cross-modal fusion strategies would be necessary for successful implementation. Exploring these multi-modal approaches remains an important avenue for advancing SAR target detection capabilities and overcoming the limitations of single-sensor systems.

Another key area for future investigation concerns the effective adaptation and application of Transformer-based architectures, potentially in hybrid configurations with CNNs, for SAR object detection. While our preliminary experiments indicated challenges in directly applying standard Transformer detectors to our current dataset scale, future research focusing on domain adaptation techniques or novel Transformer designs tailored for SAR data characteristics could yield significant breakthroughs.

Additionally, the observed class imbalance within the dataset (Figure 8) was not explicitly addressed with specific balancing techniques in this study, which might affect performance on underrepresented classes and represents an area for future improvement.

Furthermore, while the proposed NR Loss demonstrated empirical utility in addressing potential label noise for this specific dataset, a dedicated study involving rigorous theoretical analysis and broader experimental comparisons against established robust loss functions (e.g., Generalized Cross Entropy) under various noise conditions is warranted for future work to fully characterize its properties and general applicability.

In conclusion, SFEF-Net demonstrates a significant step forward in SAR aircraft detection by effectively addressing key challenges through novel architectural and loss function designs, while also highlighting promising directions for future multi-modal research.

## Figures and Tables

**Table 1:** The comparison among the traditional convolution, dilation convolution, and sparse convolution. In the ‘Illustration’ diagrams, light blue squares represent the broader sampling neighborhood, dark blue squares indicate the actual sampled positions by the kernel, and numbers 1–9 denote the specific sampled locations for typical 3 × 3 equivalent kernels (fixed for Traditional/Dilated, randomized example for Sparse).

Method	Traditional Convolution	Dilated Convolution	Sparse Convolution
Receptive Field	Small	Large	Large
Sample Positions	Fixed	Fixed	Flexible
Illustration	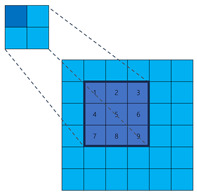	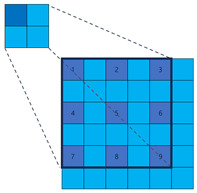	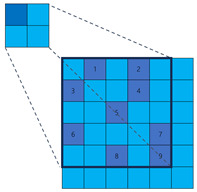

**Table 2:** The software version and experimental hyperparameters.

Parameter	Value
Epochs	500
Batch size	32
Learning rate	0.01
Momentum	0.9
Decay	0.95
Optimizer	Stochastic Gradient Descent (SGD)
Software version	Python 3.8, Pytorch 1.9

**Table 3:** Comparison of detection results between different algorithms.

Method	Precision	Recall	F1-Score	AP50	AP50-95
Faster R-CNN [22]	77.6	78.1	77.8	71.6	53.6
Cascade R-CNN [23]	89.0	79.5	84.0	77.8	59.1
RetinaNet [33]	80.1	81.2	80.6	72.3	54.1
SSD [32]	73.1	75.1	74.1	68.7	50.2
YOLOv5 [28]	88.5	81.7	84.9	85.9	59.1
YOLOv8 [31]	88.4	79.8	83.9	85.6	59.8
SA-Net [15] ^1^	87.5	82.2	84.8	80.4	61.4
SFEF-Net	**91.5**	**83.2**	**87.1**	**89.4**	**65.2**

**Bold** values indicate the best performance. ^1^ The experimental results of SA-Net are derived from [15] rather than our replication, as the paper has not yet released their source code.

**Table 4:** Comparison of model complexity and inference speed between different algorithms.

Method	AP50-95	Parameters (M)	GFLOPs	Inference Time (ms)	FPS
Faster R-CNN [22]	53.6	41.8	180.5	45.7	21.9
Cascade R-CNN [23]	59.1	69.2	260.3	68.9	14.5
RetinaNet [33]	54.1	36.7	149.6	39.5	25.3
SSD [32]	50.2	24.3	88.1	19.2	52.1
YOLOv5 [28]	59.1	7.2	16.4	4.13	242.1
YOLOv8 [31]	59.8	11.2	28.6	5.23	191.0
SA-Net [15] ^1^	61.4	—	—	—	—
SFEF-Net	65.2	8.3	18.9	4.93	202.8

^1^ The complexity (Parameters, GFLOPs) and efficiency (Inference time, FPS) metrics for SA-Net [15] are marked with ‘—’, as they could not be obtained due to the unavailability of its source code, and these specific metrics were not reported in the original paper. All other inference time and FPS values in this table were measured by us under the conditions specified in the caption (NVIDIA RTX 3090, FP16, batch size 1).

**Table 5:** Ablation study on sparse convolution.

Model	Convolution Type	Precision	Recall	F1-Score	AP50	AP50-95
YOLOv5	traditional	88.5	81.7	84.9	85.9	59.1
	sparse	**90.1**	**81.8**	**85.7**	**87.8**	**61.0**
YOLOv8	traditional	88.4	*79.8*	*83.9*	85.6	59.8
	sparse	*88.9*	79.5	*83.9*	*86.0*	*61.3*

**Bold** values indicate the best performance; *italic* values indicate the second-best performance.

**Table 6:** Ablation study on GIFD.

Neck	Precision	Recall	F1-Score	AP50	AP50-95
FPN [46]	89.4	**82.0**	85.5	87.7	60.5
PANet [47]	90.1	81.8	85.7	87.8	61.0
GIFD	**91.6**	81.9	**86.5**	**88.9**	**63.9**

**Bold** values indicate the best performance.

**Table 7:** Ablation study on NR loss.

Loss	Missing Proportion	Precision	Recall	F1-Score	AP50	AP50-95
CE Loss	0	**91.6**	81.9	86.5	88.9	63.9
NR Loss	0	91.5	**83.2**	**87.1**	**89.4**	**65.2**
CE Loss	10%	89.4	79.8	84.3	85.9	59.5
NR Loss	10%	*89.5*	*81.7*	*85.9*	*87.7*	*60.1*

**Bold** values indicate the best performance; *italic* values indicate the second-best performance.

## Data Availability

Data are contained within the article.
